# Understanding Support for European Integration Across Generations: A Study Guided by the Theory of Planned Behavior

**DOI:** 10.5964/ejop.v16i3.1844

**Published:** 2020-08-31

**Authors:** Francesco La Barbera, Icek Ajzen

**Affiliations:** a Department of Political Sciences, University of Naples Federico II, Naples, Italy; b Department of Psychological and Brain Sciences, University of Massachusetts Amherst, Amherst, MA, USA; ZPID – Leibniz Institute for Psychology Information, Trier, Germany

**Keywords:** theory of planned behavior, European studies, voting behavior, cohort differences, moderation analysis

## Abstract

Recent events, such as failed constitutional referenda, low voting turnout in the European Union parliamentary elections, and the 2016 Brexit referendum in the United Kingdom call for a better understanding of people’s voting behavior in relation to the EU. The current study is the first attempt to use the theory of planned behavior to explore the antecedents of voting for EU integration in an Italian convenience sample (N = 441) of varying age. A structural equation model of voting intentions showed an excellent fit to the data, both for the whole sample and for subsamples of young vs. old participants. Perceived behavioral control, mainly determined by participants’ beliefs about the difficulties of exerting direct democratic control through citizenship and voting, had a significant effect on intentions to vote in favor of EU integration across age groups. In addition, older people’s intentions were also affected by their attitude towards EU integration, based primarily on their beliefs about losing national identity.

In the course of the past two decades, the threat of terrorism, a deep economic recession, austerity measures, and mass migration have laid bare deep rifts among countries in the European Union (EU), rifts that threaten the viability of the European project. Management of these varied problems requires greater cooperation and deepening integration, yet Eurosceptic tendencies in several countries demand return of political and economic power from Brussels to national governments, or – in more extreme cases – exit from the EU (Rose & Borz, 2016). The 2016 Brexit referendum in the United Kingdom is the most powerful example of this trend (Hobolt, 2016). Efforts to deal with such issues require better understanding of the factors that promote development of transnational identities (see Herrmann, Risse, & Brewer, 2004; La Barbera, 2015; La Barbera, Cariota Ferrara, & Boza, 2014). Unfortunately, little research has been done to determine the psychological factors that foster or deter voting in support of EU integration. To the best of our knowledge, the study reported in this article is the first to investigate this issue in the context of a systematic and empirically validated theoretical framework, the theory of planned behavior (Ajzen, 1991 2012).

It is possible to study European integration from both a static and a dynamic perspective (Rose & Borz, 2016). The current state of the EU is the result of a process that began in the 1950’s and is characterized by centralization of power in political and economic decision-making, a partial relinquishing of national sovereignty, increased interdependence among member states, and emergence of central European institutions (Castano, 2004; La Barbera, 2015). A static approach is exemplified by public opinion surveys that ask people to evaluate aspects related to the EU as they exist today. From a dynamic perspective, however, the European project is an ongoing process aimed at an ever-closer union, as explicitly stated, for example, in the 1957 Treaty of Rome. As mentioned, however, this process of progressive integration and centralization of power runs counter to much public opinion and is far from being accepted by all member states.

General information of relevance is available in the large-scale European Election Study (EES), which measured general attitudes toward integration held by citizens of member states. Among the 27,069 participants surveyed in the 2009 European Parliament post-election survey, 34 percent were “satisfied with the EU as it is,” 33% were “in favor of further integration,” 24% thought that “integration has gone too far,” and 9% chose the “don’t know” option. Clearly, across the EU, a mere minority of citizens were in favor of further integration. In fact, only in Romania (54%) and Spain (51%) did an absolute majority of citizens line up in favor of greater integration.

The opposition to European integration is also reflected in the results of several public referenda. For example, the Treaty on European Union (TEU) was signed on February 7, 1992 by the members of the European Community in Maastricht, Netherlands, to further European integration and introduction of a single European currency. The process of ratifying the treaty was particularly difficult in Denmark, France, and the United Kingdom. In fact, the TEU was rejected in a first Danish referendum. EU integration was subsequently jeopardized by the rejection of several other important European treaty proposals, such as the Nice Treaty, the Constitutional Treaty, and the Lisbon Treaty (Hobolt, 2009). Public discontent is also evidenced indirectly by steadily decreasing voter turnout in European parliamentary elections, which has fallen from 62% in the first election in 1979 to 43% in the 2009 election, with the May 2014 EU election recording the lowest voter turnout of 42.5%. Political upheavals in several EU countries appear to have redirected attention to the European project, with voter turnout in the 2019 European elections rising to 50.9%. At the same time, however, right-wing and Eurosceptic parties have gained strength in national elections in several countries, including Germany, France, United Kingdom, Poland, and Hungary.

In Italy, the nationalist League (Lega) party’s share of the vote rose from 6% in the 2014 elections to 34% in the 2019 election^i^. Clearly, there is no universal support for greater EU integration. Instead, this is a dynamic, uncertain, and contested process in which citizens’ opinions and voting choices are gaining increasing importance. The present study addressed this dynamic aspect by examining Italian participants’ beliefs and attitudes with respect to continuing European integration.

Starting with the establishment of the European Economic Community in the 1957 Treaty of Rome, institutions have promoted integration through intergovernmental treaties that have conferred additional powers to the EU (Pollack, 2012). This centralization of power proceeded without much public input, relying on a so-called "permissive consensus" of public opinion (Haas, 1958, p. 17). However, as the EU’s impact on national policies progressively increased, and as the 2008 global economic crisis highlighted the salience of monetary integration in Euro-zone countries, citizens became increasingly aware of the interdependence of European countries and the influence of the European Union on their lives (Hobolt & Wratil, 2015). As a result, there has been a shift from passive acquiescence of the early period of integration, where leaders could make decisions without public consultation, towards a so-called *constraining dissensus*, characterized by the influence of public opinion on integration decisions (Hooghe & Marks, 2009) and by calls for greater democracy in the EU (Hobolt & Wratil, 2015): “The times when elites could pursue European integration with no regard to public opinion are long gone” (Hobolt, 2012, p. 716).

A pivotal aspect of these developments is the involvement of the general public in decision making by way of referenda that can determine the fate of further European integration (Hobolt, 2009). In the present study we applied Ajzen’s (1991, 2012) theory of planned behavior to enhance our understanding of the beliefs and attitudes that influence people’s voting decisions in such referenda.

## The Theory of Planned Behavior

The theory of planned behavior (TPB) has been used extensively to predict and explain behavior in a variety of domains: safer sex, environmental protection, exercising, dieting, and many other behaviors (see Fishbein & Ajzen, 2010, for a review). The TPB has also been employed to study and predict political behaviors, such as voting in American presidential elections (Fishbein, Ajzen, & Hinkle, 1980) and voting on health-related issues (Flynn et al.,1997; Tung, Vernick, Reiney, & Gielen, 2012). Research has shown strong and significant correlations between voting intentions and voting choice, and good prediction of voting intentions from the TPB’s main constructs (Eckstein, Noack, & Gniewosz, 2013; Hansen & Jensen, 2007). Nonetheless, the TPB has not been used so far for understanding and predicting voting intentions in relation to the EU integration process.

When applied to support for EU integration, the TPB posits that a vote for (or against) a proposition to promote integration is preceded by the intention to vote for (or against) it. To understand the voting decision, it is therefore necessary to explore the factors that determine this intention. According to the TPB, three factors influence the intention to vote in support of European integration: attitude toward this behavior (ATT); perceived social pressure to vote in favor or against integration, or subjective norm (SN); and perceived behavioral control (PBC). While attitudes and subjective norms with respect to voting in favor of European integration are easily conceptualized and measured, the question of perceived behavioral control requires elaboration. Citizens clearly have control over their voting behavior; i.e., they can easily participate in elections and vote for or against a proposed policy. Perceived control, in this context, has largely to do with the perceived ease or difficulty of making an informed decision in relation to such a vote (Fishbein & Ajzen, 2010).

Direct measures of ATT, SN, and PBC provide only a broad outline of the factors that determine the decision to vote in favor of EU integration. To obtain a more fine-grained understanding, we must explore the specific considerations that underlie ATT, SN, and PBC. According to the TPB, the attitude toward voting in favor of EU integration is determined by beliefs about the likely consequences of doing so (*behavioral beliefs*). The more favorable the perceived consequences of EU integration, the more positive the attitude will be. Subjective norms, in turn, are determined by *normative beliefs,* i.e., beliefs that important others would approve or disapprove of a vote in favor of greater integration, or that these social referents themselves are likely to vote in favor of or against integration. Finally, PBC is a function of *control beliefs*, i.e., beliefs about the presence of factors that may facilitate or impede an informed vote for or against EU integration. Examination of these beliefs can not only help to explain people’s support for, or opposition to, EU integration, it can also guide intervention strategies designed to enhance support.

In addition to exploring the theoretical antecedents of voting intentions in regard to European integration, our study also examined age-related differences in these intentions. Previous research has shown a significant influence of age on identification with the EU, attitude towards EU integration, and willingness to vote in favor of the EU, but the results of this research have not been entirely consistent. For example, in a study involving a representative Dutch sample, Boomgaarden, Schuck, Elenbaas, and de Vreese (2011) found a significant positive correlation between age and attitude towards the EU and identification with the EU: the older the participants, the more favorable were their attitudes and the higher was their level of identification with the EU. At the same time, age has also been shown to predispose voting against the EU. With respect to the Brexit referendum, for example, a 50-year-old voter was 10% more likely to support the “Leave” option compared to a 33-year-old (Hobolt, 2016).

Complicating matters is the distinction between cohort effects and age effects (Lutz, Kritzinger & Skirbekk, 2006). Cross-sectional studies that compare sub-samples varying in age can only reveal cohort effects whereas longitudinal data also permit examination of changes over time. In a longitudinal study on multiple identities, Lutz et al. (2006) found a positive cohort effect such that for every year born later, the proportion of participants who identified with the EU as well as with their own nation increased by roughly 0.5%, compared to those who identified only with their own nation. Secondary in size was an age effect across time, which showed that dual identities reached a peak around age 50-60 and then started to decline. Overall, the results suggest a relatively weak curvilinear relation between advancing age and identification with the EU, and a strong negative cohort effect, such that older generations are less identified with the EU than more recent generations. Assuming that the estimated effects will continue, the authors estimated that in 2030 there will be about 100 million EU citizens who have strictly national identities, compared to more than 220 million with multiple identities that include identification with the European Union.

The present study explored the basis for the strong cohort effect. Specifically, we used the TPB to examine differences due to respondents’ age in intentions to vote in favor of EU integration, as well as differences in beliefs and attitudes that determine those intentions.

## Aims of the Current Research

The major aim of this study was to establish the predictive power of attitude, subjective norm, and perceived behavioral control in relation to the intention to vote in favor of EU integration. A second objective was to identify the key behavioral, normative, and control beliefs that provide the basis, respectively, for attitudes, subjective norms, and perceived behavioral control. A third aim was to test the role of age as a moderator of the effects^ii^ of the three major TPB constructs (ATT, SN, PBC) on intention, and of the effect of beliefs on these three major constructs.

In order to explore the role of age in this domain, we examined the moderating effects of age on a) the relations between the TPB antecedents and intention and b) the relations between behavioral, normative and control beliefs and ATT, SN, and PBC, respectively.

## Method

### Pilot Study

Relying on the guidelines provided by Fishbein and Ajzen (2010, Appendix), we conducted a pilot study in which we elicited readily accessible beliefs about voting in favor of EU integration using an open-ended questionnaire. The pilot sample consisted of 100 respondents (58 females; age range 18-69, *M* = 35.02, *SD* = 11.73) recruited in public buildings in Italy (e.g., university buildings, post offices). As suggested by La Barbera (2015), EU integration was defined for the participants in terms of five key aspects: (1) The EU becoming a single country; (2) centralized management of the European economy to replace the economic powers of individual states; (3) direct, unmediated implementation of EU directives in member states; (4) a single EU army to replace national armies; (5) more political power to the EU, greater than the power of each single country. They were then asked to take a few minutes to write down the thoughts that came to mind about voting in favor of EU integration, as defined. Specifically, they were asked to list what they thought would be the advantages and disadvantages of voting in favor of EU integration, the people and/or groups who would approve or disapprove of their voting in favor of EU integration, and the factors that could facilitate or hinder their voting in favor of EU integration.

A content analysis of the responses was conducted to determine the most frequent themes in terms of behavioral outcomes, normative referents, and control factors. Three researchers jointly analyzed participants’ written responses to the open-ended questions and the interpretations required consensus of all three. This process resulted in a classification of the individual responses listed into common categories (Galli, Fasanelli, & Schember, 2018). The most frequent responses, cited by at least 15% of the participants, were used to construct modal sets of behavioral, normative, and control beliefs included in the main study described below.

The six most frequently mentioned likely outcomes of voting in favor of EU integration were a centralized economy, losing national identity, increased job opportunities, more cultural exchange, more transparent politics, and an emerging EU identity. The four modal normative referents that emerged from the content analysis were family (relatives), political groups, colleagues at work, and friends. Finally, the four most frequently mentioned control issues for making an informed decision were insufficient information, not always being considered an EU citizen, a deficit of democracy, and misinformation.

### Main Study

#### Procedure

Participants for the main study were contacted in public buildings in Italy. A total of 500 questionnaires were administered to a convenience sample; 59 participants were dropped because they failed to complete and/or return the questionnaire. The final sample consists of 441 participants (235 females, aged 18 to 71, *M*_age_ = 36.9, *Mdn*_age_ = 32.00, *SD*_age_ = 13.68), who completed the questionnaire described below. The survey was conducted in Italian. The items described in this paper are translations from the Italian (the original Italian items can be obtained by writing to the first author). The items in the questionnaire referred to EU integration, which was explicitly defined as an ongoing process in terms of the same five key points used in the pilot study. Multiple items for each main construct were aggregated into a single score for computing correlations among the constructs, whereas each item was used as an indicator of the corresponding latent construct in the SEM.

#### Questionnaire

##### Behavioral beliefs

In the TPB, an expectancy-value model describes the relation between accessible behavioral beliefs and attitude toward the behavior (see Ajzen, 1991; Fishbein and Ajzen, 2010). Specifically, attitude toward voting in favor of EU integration (the behavior) is assumed to be determined by the total set of accessible behavioral beliefs linking the behavior to various expected outcomes. The strength of each belief (b) is weighted by the evaluation (e) of the outcome, and the products are aggregated, as shown in the following equation:

Therefore, participants were asked to rate the likelihood that voting in favor of EU integration would produce each of the six outcomes identified in the pilot study (for example, losing national identity) on a 7-point scale ranging from “*extremely unlikely*” to “*extremely likely*.” In addition, they rated each outcome on a 7-point scale from “*extremely negative*” to “*extremely positive*.” For each behavioral belief, we obtained a score by multiplying the rated likelihood of the outcome by its subjective value, and then summing the product scores across all beliefs.

The same procedure was used to obtain the composite scores of normative beliefs and control beliefs described below.

##### Normative beliefs

To measure normative beliefs, participants were asked to indicate to what extent they thought that the four important referents identified in the pilot study would approve of their voting for EU integration on 7-point scales ranging from “*very unlikely*” to “*very likely*.” Participants were also asked about the importance of each of these referents on 7-point scales ranging from “*not at all*” to “*very much*.” To obtain a score for each normative belief we used the same procedure described in relation to behavioral beliefs. The higher the score, the stronger is the perceived influence of the normative referent.

##### Control beliefs

Participants were asked how frequently they thought about the existence of the four control factors that emerged in the pilot study (e.g., having sufficient information for an informed decision). Answers were collected on 7-point scales ranging from “*very rarely*” to “*very frequently*.” They also were asked to evaluate, for each control factor, its power to facilitate-impede performance of the behavior on 7-point scales. As before, a control belief measure was obtained by multiplying the two scores. The resulting products were then recoded so that the higher the score, the higher the perceived control in relation to the particular control factor.

##### Intention

To assess participants’ intentions to vote in favor of greater EU integration, we used five items corresponding to the five enumerated aspects of integration (e.g., “Would you vote for making the EU a single country?”). Answers were collected on 7-point scales ranging from “*definitely not*” to “*yes, definitely*,” and then averaged across items to produce a single composite score, with higher values indicating a more favorable intention to vote for EU integration (Cronbach’s α = .84).

##### Attitude

Attitudes towards voting in favor of EU integration were assessed by asking participants to rate “For me, voting in favor of European integration is:” on two 7-point bipolar adjective scales: “*good* – *bad*” (reverse coded) and “*unpleasant* – *pleasant*.” Responses were aggregated into a composite measure by averaging the scores on the two scales (Spearman-Brown Rho = .72). Higher values indicate more positive attitudes.

##### Subjective norm

Two items were used to measure subjective norm: “Most people who are important to me believe that I should vote in favor of European integration” and “Others expect me to vote in favor of European integration.” Participants answered on 7-point scales ranging from “*definitely not*” to “*yes, definitely*.” The answers were again aggregated into a single score by computing the mean across the two scales (Spearman-Brown Rho = .62). Higher values indicate greater perceived social support for voting in favor of European integration.

##### Perceived behavioral control

Two items were used to measure perceived behavioral control: “For me the act of voting in favor of European integration is: (1) impossible-possible and (2) difficult-easy” (Spearman-Brown Rho = .64). Responses on the 7-point scales were averaged. Higher values indicate higher perceived control.

#### Statistical Analyses

Structural equation modeling (SEM) was used (by STATA 15) to test the significance and power of the main TPB constructs (ATT, SN, and PBC) to predict intention to vote in favor of EU integration. Model fit was assessed with the comparative fit index (CFI), the Tucker-Lewis index (TLI), and the root mean squared error of approximation (RMSEA). According to conventional rules of thumb (Hu & Bentler, 1999; Kline, 2011), acceptable and excellent model fit are indicated by CFI and TLI values greater than .90 and .95, respectively, and by RMSEA values smaller than .08 and .06, respectively. After testing for measurement invariance across age-based groups, we investigated whether age moderated the relations between the three exogenous factors (ATT, SN, PBC) and intention. In addition, in order to ascertain if the beliefs we selected adequately represented the latent constructs, we estimated multiple indicators, multiple causes (MIMIC) models (Kline, 2011) in which, in line with the TPB, each latent construct is *formed* by beliefs and is *reflected* in the answers to direct items. Finally, we assessed whether age moderated the relations between behavioral, normative, and control beliefs and their respective latent constructs.

Because we were interested in cohort effects, rather than in changes over time (Lutz et al., 2006), we used a median split between well distinguishable age groups to examine differences in the antecedents of intention (ATT, SN, PBC), and in the beliefs underpinning these antecedents.

## Results

Descriptive statistics for the direct measures of ATT, SN, PBC, and INT are provided in Table 1. In the 441 (88%) retained questionnaires, the frequency of missing data was quite low (see Table 1). The STATA 15 MLMV (Maximum Likelihood with Missing Value) estimation routine was used to deal with missing data.

**Table 1:** TPB Items’ Means, Standard Deviations, and Missing Data

Item	*M*	*SD*	Missing data	%
Intention - EU single country	4.09	2.10	2	0.45
Intention - centralized economy over state sovereignty	3.98	1.99	1	0.23
Intention - immediate application norms	4.99	1.81	3	0.68
Intention - single army	4.20	2.05	1	0.23
Intention - more political power than states	3.97	1.97	0	0
Attitude - good/bad (reverse coded)	5.03	1.71	5	1.13
Attitude – unpleasant/pleasant	4.61	1.64	21	4.76
Norms – others	4.65	1.60	1	0.23
Norms – most people I care	4.45	1.52	0	0
PBC – difficult/easy	4.60	1.69	0	0
PBC – impossible/possible	5.08	1.64	2	0.45

*Note.* PBC = Perceived behavioral control.

Table 2 displays means and standard deviations of the study variables as well as correlations among these variables. Intention correlated significantly with direct measures of attitude, subjective norm, and perceived behavioral control, as well as with all belief composites. The correlations ranged from .129 for normative beliefs to .283 for PBC. In addition, and as expected, attitude, subjective norm, and PBC correlated significantly with their corresponding belief composites. The attitude-behavioral belief composite correlation was .412, the correlation between subjective norm and the normative belief composite was .169, and control beliefs correlated .108 with PBC.

**Table 2:** Correlations, Means and Standard Deviations of Study Variables’ Aggregate Scores

Variable	1	2	3	5	6	7	8
1. Intention	4.25^a^ (1.56)						
2. ATT	.277***	4.81^a^ (1.48)					
3. SN	.189***	.453***	4.56^a^ (1.30)				
5. PBC	.283***	.437***	.260***	4.84^a^ (1.43)			
6. Behavioral beliefs	.264***	.412***	.364***	.250***	24.21^b^ (8.64)		
7. Normative beliefs	.129**	.060	.169***	-.026	.127*	13.49^b^ (7.90)	
8. Control beliefs	.237***	.267***	.198***	.108*	.282***	-.101*	25.38^b^ (10.18)

*Note.* The table shows Pearson’s *r* correlation coefficients. Diagonal cells report the variable mean (standard deviation in parentheses). ATT = Attitude; SN = Subjective norm; PBC = Perceived behavioral control.

^a^range 1-7. ^b^range 1-49.

*p < .05. **p < .01. ***p < .001.

In order to perform the multigroup analyses, the age distribution was split at the median, creating a dichotomous variable with 0 (below median) representing younger people, and 1 (above median) the older ones. Table 3 provides a summary of the two age-based groups’ characteristics. Looking at means and medians, the two subgroups represent well distinguishable age clusters, roughly identifiable with baby boomers and generation X (older group) and millennials (younger group). The two age-based groups are well balanced across gender and education (Chi squared tests were not significant for either variable, *p*s > .05).

**Table 3:** Age-Based Group Characteristics

Variable	Younger (*n* = 223)	Older (*n* = 218)
Age		
*M* (*SD*)	24.91 (3.12)	49.18 (8.37)
Gender		
Male	95	111
Female	128	107
Education		
Undergraduates	143	154
College education	80	64

The between-group differences in relation to the study variable means were also tested. Attitude and perceived behavioral control were higher for older participants (*M*_ATT_ = 4.96, *SD* = 1.45; *M*_PBC_ = 5.03, *SD* = 1.39), compared to younger participants (*M*_ATT_ = 4.67, *SD* = 1.50; *M*_PBC_ = 4.65, *SD* = 1.44), *t*(417) = 2.036, *p* < .05 and *t*(437) = 2.831, *p* < .01. Differences in intention and subjective norm scores across groups were non-significant (*t*s < 1).

### Structural Equation Modeling

**Figure 1:**
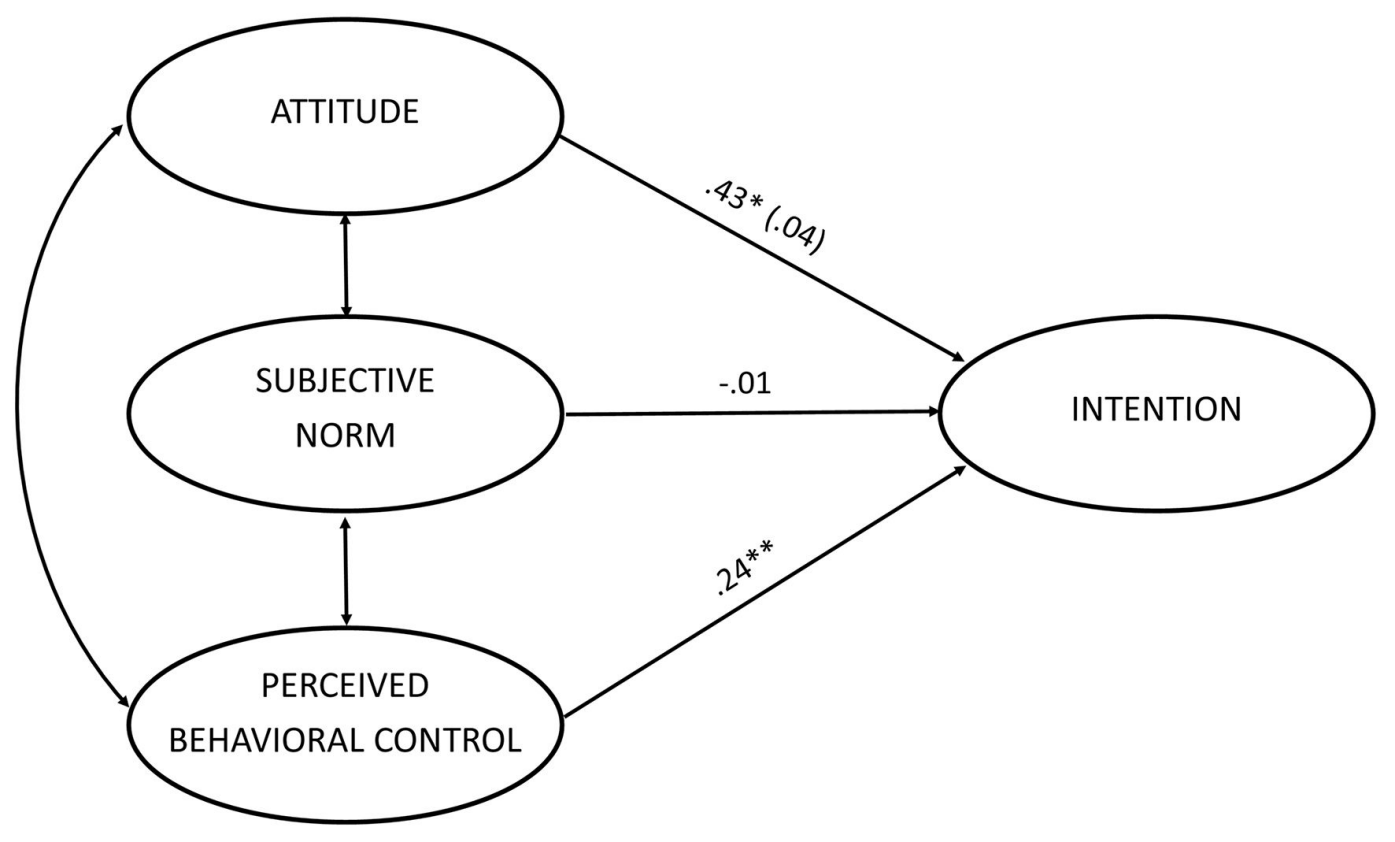
TPB model of the intention to vote for EU integration. *Note.* Age significantly moderated the effect of attitude on intention, whereas it was not a significant moderator of the influence of SN and PBC. Therefore, for attitude, different coefficients for the older and younger participants are reported (younger participants in parentheses). A single coefficient is reported for SN and PBC. *p < .05. **p < .01.

The TPB model, in which intentions are predicted from attitude, subjective norm, and perceived behavioral control (Figure 1) showed an excellent fit to the data for the entire sample, CFI = .960, TLI = .944, RMSEA = .052. The model accounted for 39% of the variance in intentions. Structural coefficients showed that intention to vote for EU integration was significantly affected only by perceived behavioral control (β = .24, *p* = .01). The influence of attitude was marginal (β = .20, *p* = .10) whereas subjective norms did not significantly affect intention (*z* < 1).

Multigroup structural equation modeling was used to assess measurement invariance across age groups, using chi-square difference tests for a set of nested models. Complete results are provided in Table 4. The fit of the configural model (Model 1) was excellent, suggesting that the factor structure was adequate in both groups. To test metric invariance, in Model 2 we constrained factor loadings to be equal across groups, that is, the parameters that represented factor loadings were not allowed to vary across age-based groups. The chi-square test showed a non-significant difference between this model and the baseline model, indicating that factor loading differences between groups were not significant. In Model 3, to test strong (scalar) invariance, intercepts were constrained to be equal across groups. Model 3 did not yield a significant chi-square difference compared to Model 2, thus supporting scalar invariance across groups. Finally, in Model 4, a constraint on residuals was added for testing strict measurement invariance across groups. Compared with the previous model, Model 4 did not a show a significant difference. Thus, the invariance tests showed that our measurement model had a satisfactory fit for both groups. Also, the model comparison based on CFI and RMSEA (see Table 4) supports strict measurement invariance across age-based groups in our data (Cheung & Rensvold, 2002). The multigroup model accounted for 66% of the variance in intentions for the entire sample.

**Table 4:** TPB-Based Structural Equation Models of Intention to Vote for EU Integration

Model	Model fit				Model comparison			
	χ^2^	*df*	TLI	CFI	RMSEA	Comparison	Δχ^2^	Δ*df*
Model 1	103.55	76	.972	.980	.041			
Model 2	111.04	83	.974	.980	.039	M2-M1	7.49	7
Model 3	120.20	91	.975	.979	.038	M3-M2	9.17	8
Model 4	133.11	102	.976	.978	.037	M4-M3	12.91	11
Model 5	138.56	105	.975	.976	.038	M5-M4	5.45	3
Model 6	133.71	104	.978	.979	.036	M5-M6	4.84*	1
Model 7	136.49	104	.976	.977	.038	M5-M7	2.07	1
Model 8	135.86	104	.976	.977	.037	M5-M8	2.69	1

*Note*. TLI = Tucker-Lewis index; CFI = Comparative fit index; RMSEA = Root mean squared error of approximation.

*p < .05.

In Model 5, we tested the moderation hypotheses by constraining the structural coefficients to be equal across age groups. The model fit did not differ significantly from the previous model, thus suggesting that the overall magnitude of the direct path coefficients was comparable across the age-based groups. This may also indicate that not all structural path coefficients were different across age-based groups (Neff, 1985). Therefore, we tested three additional models regarding the moderating effect of age on the relations between attitude, subjective norms, and perceived behavioral control on one hand and intention on the other (Sauer & Dick, 1993; see also Kenny, 2011). In Model 6, we unconstrained the path from attitude to intention, and the Chi-square test showed a significant difference. Indeed, the structural coefficients indicated that intention to vote for EU integration was significantly affected by attitude towards EU integration only in the case of older people (β_old_ = .43, *p* < .05; β_young_ = .04, *z* < 1).

The paths from subjective norms and perceived behavioral control to intention were unconstrained in Models 7 and 8, respectively. The chi-square tests showed a non-significant difference for both. Table 4 provides complete results of these comparisons.

In sum, the multigroup analysis showed that the effect of attitude on intention was qualified by age, whereas moderation by age was not significant for SN and PBC. The results of the analyses performed for the whole sample and across age-based groups are summarized in Figure 1.

### Behavioral, Normative, and Control Beliefs

Next, we examined the individual behavioral and control beliefs that had a significant impact on the corresponding latent constructs. We identified the key beliefs for each factor across age-based groups. We did not perform this analysis for subjective norm because it did not exert a significant effect on intention in any of our previous analyses. Descriptive statistics of beliefs across groups are provided in Table 5. No significant differences emerged between older and younger participants except for the behavioral belief regarding political transparency, *t*(453) = 2.25, *p* = .025, with higher mean scores for older as compared to younger people. The difference related to losing national identity and emerging of an EU identity were significant at the 10% level.

**Table 5:** Descriptive Statistics of Beliefs Across Age-Based Groups

Group	*M*	*SD*
Centralized economy		
Young	19.65	10.82
Old	21.41	12.25
Losing national identity		
Young	20.42	12.91
Old	18.36	11.70
More job opportunities		
Young	26.46	14.23
Old	27.82	14.71
Cultural exchange		
Young	29.25	16.04
Old	30.14	16.34
EU identity		
Young	21.78	11.85
Old	24.11	13.45
Political transparency		
Young	23.56	12.48
Old	26.35	13.43
More information		
Young	23.69	13.62
Old	24.26	13.03
Misinformation		
Young	26.25	13.11
Old	25.72	12.30
European citizenship		
Young	26.31	13.50
Old	27.25	13.68
Democratic deficit		
Young	24.96	13.66
Old	24.10	12.64

We ran a multigroup (younger vs. older participants) MIMIC model in which attitude is *formed* by the six beliefs we identified in the pilot study and is *reflected* in the two items we used in previous models as a direct measure of attitude. The goodness of fit of the model was excellent: CFI = .98, TLI = .96, RMSEA = .040. Next, we constrained all structural coefficients to be equal across groups. This model also had an excellent fit to the data, and the Chi Square difference test showed a marginally significant difference between this model and the previous model with unconstrained structural coefficients, χ^2^(6) = 11.22, *p* = .08. Then, we compared six different models, each with a single path free to vary across age groups, against the model with all structural coefficients constrained. Results are provided in Table 6. The models in which we unconstrained the beliefs about losing national identity and cultural exchange were statistically different compared to the constrained model. The model in which the belief about EU fostering centralized economy was unconstrained across groups also reached a marginally significant difference compared to the reference model. These three beliefs affected attitude only for older participants. Finally, there was some suggestion that younger participants’ attitudes were affected by beliefs about EU fostering more job opportunities and more transparent policy, even if this was not fully supported by the Chi square difference tests.

**Table 6:** The Effect of Behavioral Beliefs on Attitude by Age

Belief	Group	β	*SE*	Δχ^2^
Voting for European integration will foster a centralized economy	Younger	-.02	.08	3.42^†^
	Older	.16^†^	.09	
Voting for European integration will mean a loss of national identity (reverse coded)	Younger	.04	.06	3.67*
	Older	.14*	.06	
Voting for European integration will mean more job opportunities	Younger	.37**	.09	1.40
	Older	.17^†^	.10	
Voting for European integration will foster cultural exchange	Younger	-.07	.09	4.11*
	Older	.18^†^	.09	
Voting for European integration will foster the birth of an European cultural and political identity	Younger	.13	.08	1.97
	Older	.14^†^	.08	
Voting for European integration will mean more transparent policy	Younger	.16*	.08	0.96
	Older	.01	.08	

*Note.* In each model a single path is unconstrained (corresponding to a specific belief), and the model is compared against the one in which all structural coefficients are constrained across groups. Results of the χ^2^ difference tests are reported in the last column. All *df* = 1.

*p < .05. **p < .01. ^†^*p* < .10.

We also ran a multigroup MIMIC model for perceived behavioral control, and the goodness of fit was excellent: CFI = 1, TLI = 1, RMSEA < .001. In the subsequent model, all structural coefficients were constrained to be equal across groups. This model had an excellent fit to the data, and the Chi Square test showed a marginal difference between this and the previous model, χ^2^(4) = 9.60, *p* = .05. Four additional models were performed, each with a single path unconstrained, and we compared this against the model in which all structural coefficients were constrained across groups. Results are provided in Table 7. It can be seen that all unconstrained models were statistically different compared to the constrained model.

**Table 7:** The Effect of Control Beliefs on PBC Across Age-Based Groups

Belief (all reverse coded)	Group	β	*SE*	Δχ^2^
Not having enough information to vote in favor of European integration	Younger	-.08	.10	9.27**
	Older	.05	.10	
Disinformation towards European integration	Younger	.11	.09	7.62**
	Older	.10	.09	
Not always being considered a European citizen	Younger	.19*	.09	8.19**
	Older	.23*	.10	
“Democratic deficit” in the EU	Younger	.15*	.07	6.22*
	Older	.21**	.07	

*Note.* In each model a single path is unconstrained (corresponding to a specific belief), and the model is compared against the one in which all structural coefficients are constrained across groups. Results of the χ^2^ difference tests are reported in the last column. All *df* = 1.

*p < .05. **p < .01.

Inspection of the path coefficients revealed that two beliefs—the idea about not being considered a European citizen and about the presence of a democratic deficit in the EU—exerted a significant influence on perceived control for both age-based groups, although it was slightly larger in the case of older people.

## Discussion

The aims of the current study were to test whether a TPB-based model could predict intentions to vote for EU integration and to explore the factors underlying voting choice. Results provide, for the first time, support for a TPB-based model of voting in favor of EU integration, showing an excellent fit to the data. Importantly, the invariance test showed that the basic structure and the main measurement characteristics of the TPB model were comparable for the two age-based groups of participants, although they belong to different generations.

Among the major constructs of the TPB, attitude and perceived behavioral control were of particular importance as predictors of intentions, while the relative contribution of subjective norms was not significant. This could be due to the fact that, in our study, only injunctive social norms were measured. Previous research has shown that including descriptive norms can increase the predictive power of this variable; also, measures designed to tap into social identification can add important information regarding the importance of subjective norms (see White, Smith, Terry, Greenslade, & McKimmie, 2009, for a review). Therefore, future studies could further explore the normative dimension for a more complete understanding of its relation to intentions to vote in favor of EU integration.

Perhaps of greatest interest, we found that the prediction of intentions from attitudes was qualified by age, as revealed by a multi-group analysis. This analysis supported a clear distinction between older and younger participants. Generally speaking, only the intentions of older people to vote in favor of EU integration were related to their attitudes, and these attitudes were mainly related of their beliefs about losing national identity. By comparison, the intentions of younger participants were related only to perceived control, in particular to their beliefs about the difficulties of exerting direct democratic control through citizenship and voting.

This pattern of findings may reflect the fact that older participants, largely between 40 and 60 years of age, belong to generations whose political socialization (van Deth et al., 2011) took place when the European project was “under construction,” involving various stages of transition (MEC, CEE, EU). They experienced the emergence of a super-national identity, the important event of the Maastricht Treaty, free movement of people across national borders and, especially, the introduction of a single currency. The introduction of the Euro was an event of historical significance with practical as well as symbolical implications involving the loss of national currency and the “reification” of the new super-national entity: “The Euro makes Europe real” (Risse, 2003, p. 487). These two innovative aspects of the integration process are reflected in our findings: Results of the MIMIC model highlight the importance of the beliefs about cultural exchange and a centralized economy in relation to the attitudes of older participants. Our younger participants, in contrast, belong to a generation whose political socialization took place when the EU and the Euro were already in place. Many in this generation see themselves as EU citizens who evaluate the EU from within, as members of a political community, and their attitudes rest on their beliefs about opportunities that the EU can provide, more than on the threat to national identity (Bruter, 2008).

These two points—the importance attributed to opportunities and the low impact of identity threat—are also reflected by the MIMIC model results. The use of MIMIC model was crucial for understanding the different weight of each belief in relation to the corresponding latent construct and across groups, especially because there were no significant differences between the mean scores of younger and older participants (except that for one behavioral belief). Research is often very interested in mean differences between individuals’ beliefs, measured by self-report instruments. Of course, the difference between individuals or groups as regards a certain psychological belief should not be confused with the difference in the effect that a psychological belief exerts on a dependent variable or a latent construct (e.g. attitude) in the case of different individuals or groups (Scafuto & La Barbera, 2016). The use of multi-group MIMIC models, as in our study, allowed us to 1) evaluate, with a single formal test, how well a latent construct is *represented* by a set of indicators and *formed* by a set of items theoretically or empirically determined; 2) assess the relation of each of those elements to the latent construct; and 3) test the difference between groups regarding the previous two points.

All of the six identified behavioral beliefs showed a significant relation to attitude, at least for one of the age-based groups. Two of the four control beliefs related significantly to PBC across both age groups. The MIMIC models that included attitude, perceived behavioral control and their respective beliefs showed an excellent fit to the data. Taken together, these findings support the hypothesis that the beliefs we elicited and selected in the pilot study, and then measured in the main study, are major determinants of the latent constructs which, in turn, affect intention.

Finally, our findings demonstrate the role of moderators in TPB-based studies, and the importance of carrying out multigroup analyses. The TPB not only provides a clear depiction of the factors influencing intentions and behaviors in the framework of a general theory, it also offers the opportunity to understand the effects of individual and contextual background factors potentially relevant in relation to a specific behavior. As Lewin pointed out in 1946: “It is important to understand clearly that social research concerns itself with two rather different types of questions, namely the study of general laws and the diagnosis of specific situations. [...] For any field of action both types of scientific research are needed.” (Lewin, 1946, pp. 36-37). Our moderation analyses revealed different beliefs underpinning TPB constructs for different age groups. Without a multigroup analysis, we would have considered the effect of attitude on intention to be non-significant, producing a misunderstanding of results. Unfortunately, relatively few studies have carried out moderation analyses regarding the relations between the TPB constructs and intentions (e.g., Carfora, Caso, Sparks, & Conner, 2017), and regarding the relations between the TPB constructs and the underpinning beliefs (e.g., de Leeuw, Valois, Ajzen, & Schmidt, 2015). Our results support the idea that evaluating moderating variables can foster a more comprehensive understanding of people’s intentions and consequently can also provide a basis for more effective behavior change interventions.

Indeed, from a practical point of view, several recommendations may be derived from our findings. First, the idea of the EU threatening national identity seems to be still a concern for older people. This is in line with the rise of Euro-sceptic and/or nationalistic movements in several European countries, as mentioned in the introduction. Nationalism is a strong obstacle to identification with the EU, undermining further integration (Cinpoes, 2008). This is also in line with a rich tradition of studies about the relations among national identity, European identity, and support for EU integration (Kranendonk, Vermeulen, & van Heelsum, 2018). Field studies as well as experiments (e.g., La Barbera, 2015; La Barbera & Cariota Ferrara, 2012; La Barbera, Cariota Ferrara, & Boza, 2014) have shown that identification with the EU exerts a significant positive effect on support for EU integration. The relation between national and European identity is less clear. Early research found a negative relation between the two types of identity (Cinnirella, 1997), whereas more recent research has shown that they can coexist or even be positively correlated (Bruter, 2003, 2009). The finding that national and European identities coincide in some cases but are contradictory in others (Risse, 2010) suggests the presence of moderators (Hooghe & Marks, 2005), a topic worthy of further study.

EU institutions have tried to communicate their interest in preserving national identities, even in the most important formal acts (e.g., “The Union shall respect the national identities of its Member States”, Treaty on European Union, Article 6.3). Nonetheless, the perception of the EU as a threat to national identity still seems to be alive, particularly among older people whose attitudes and voting intentions reflect their concern about loss of national identity (see also Rippl & Seipel, 2012; Hobolt, 2016). It follows that communication strategies designed to foster support for greater EU integration should rely on messages designed to reduce or mitigate this fear. For example, experimental cross-national studies have found that framing the EU as a common project tends to foster support for EU integration more than framing the EU as common heritage (La Barbera, 2015). Because we found that support for EU integration and beliefs about losing national identity are inversely correlated, it would be interesting to investigate whether a common project framing is effective in reducing the national identity threat perception. Also, the use of EU-related news and symbols has been shown to have a dramatic impact on local and European attitudes, identity, and behaviors (Bruter, 2003; Guéguen, Martin, & Stefan, 2017), a topic worthy of further investigation.

Future support for EU integration depends in large measure on the views of young people who will ultimately determine the fate of the European project, and it is therefore this generation that is the most relevant target for institutional communications. Our findings show that threat to national identity was not a major factor for our younger participants. This is in line with findings that compared to older cohorts, younger cohorts are and will continue to be more multi-identified with their own nations and with the EU (Lutz et al., 2006). For the current study’s younger participants, intention to vote for EU integration was affected solely by perceived behavioral control. This might suggest that institutional communications aimed to changing attitudes may not be an effective way to foster pro-EU intentions and behaviors among the young. Different strategies, such as promoting European trans-national mobility (Mazzoni et al., 2018; Stoeckel, 2016), could be more effective in raising perceived control, EU-citizenship and pro-EU voting intentions.

In sum, the issue of greatest importance has to do with political representativeness. The EU faces a problem of perceived legitimacy, which can be addressed by strategies designed to enhance the perceived connection between voting in Europe-wide elections and central political processes (Arkan, 2013; Hobolt, 2012; Hobolt & Wratil, 2015). In addition, EU institutions should emphasize the value of political participation as a means toward greater representation and as the best way to exert one’s own citizenship rights. Such an approach could foster greater perceived control over effective participation, which we found to be an important determinant of voting in favor of EU integration. It stands to reason that a greater sense of being able to exert influence on European policies can be an effective way to alleviate the problems of legitimacy, thereby increasing support for European integration.

The current study has several limitations, which need to be discussed. First, the convenience sample employed implies that our findings may not be representative of the Italian population at large. In addition, our results may be limited to the Italian context. Future research should involve cross-national comparisons in order to examine the generalizability of our findings. Second, direct measures of latent constructs may have been influenced by the items about beliefs that preceded or followed them in the questionnaire. Even if past research has not found consistent results regarding the effect of different item orders (Fishbein & Ajzen, 2010), we did not examine this issue in our study. Third, the study did not consider potentially relevant background factors. Political orientation of participants, for example, could be an important variable to be considered by future research, to foster a more complete understanding of different attitudes regarding EU integration, and the beliefs that underpin them. Finally, EU integration is a complex process that involves several dimensions, including but not limited to economy, military, and immigration policies. However, research has commonly studied psychological constructs (e.g. attitude, identification) only in relation to EU integration as a whole, without distinguishing between different dimensions. Our study is in line with this literature, but it would be also important to conduct studies on more focused aspects, or comparing dimensions, for example, comparing the intention to vote in favor of economic integration vs. military unification.
